# Undisclosed cocaine use and chest pain in emergency departments of Spain

**DOI:** 10.1186/1757-7241-17-11

**Published:** 2009-03-02

**Authors:** Guillermo Burillo-Putze, Beatriz López, Juan María Borreguero León, Miquel Sánchez Sánchez, Martin García González, Alberto Domínguez Rodriguez, Eva Vallbona Afonso, Alejandro Jiménez Sosa, Oscar Mirò

**Affiliations:** 1 Emergency Department, Hospital Universitario de Canarias, La Laguna, Santa Cruz de Tenerife, Tenerife, Spain; 2 Emergency Department, Hospital Clínic, Barcelona, Spain; 3 Cardiac Intensive Care Unit, Hospital Universitario de Canarias, La Laguna, Santa Cruz de Tenerife, Tenerife, Spain; 4 Research Unit, Hospital Universitario de Canarias, La Laguna, Santa Cruz de Tenerife, Tenerife, Spain

## Abstract

**Aims:**

Illicit cocaine consumption in Spain is one of the highest in Europe. Our objective was to study the incidence of undisclosed cocaine consumption in patients attending in two Spanish Emergency Departments for chest pain.

**Methods:**

We analysed urine samples from consenting consecutive patients attending ED for chest pain to determine the presence of cocaine, and other drugs, by semiquantative tests with fluorescence polarization immunoassay (FPIA).

**Results:**

Of 140 cases, 15.7 presented positive test for drugs, and cocaine was present in 6.4%. All cocaine-positive patients were younger (p < 0.001); none was admitted to Hospital (p = 0.08). No significant differences in ED stay or need for hospitalization were found between cocaine-positive and negative patients.

**Conclusion:**

This finding in chest pain patients who consented to urine analysis suggests that the true incidence of cocaine use leading to such ED visits may be higher.

## Introduction

Illicit cocaine consumption in Spain is, together with the United Kingdom, the highest in Europe, mainly in young people [1,2].

The relationship between cocaine use and episodes of coronary ischemia or chest pain is clear, and cocaine is considered a new risk factor for cardiovascular events in chronic users [3-5].

To our knowledge, few studies have been performed in Spain on the prevalence of cocaine consumption in patients seeking Emergency Department attention when this was not the direct reason for the visit [6,7].

The objective of this work was to study the incidence of undisclosed cocaine consumption in patients attending the Emergency Department (ED) of two hospitals for chest pain.

## Patients and methods

Between May and June 2006, we prospectively studied urine samples from consecutive patients over 18 years who were attended at two University Hospitals Emergency Departments (Tenerife, Canary Islands and Barcelona, Catalonia) for non-traumatic chest pain of probable cardiovascular origin, initially not-related with cocaine consumption.

Barcelona Hospital Clinic is a large, inner city, university tertiary-care hospital, with a specialised Chest Pain Unit within its ED. All Barcelona patients included in this study were ED patients with chest pain attended by this unit. The University Hospital of the Canary Islands (HUC) is a large, suburban, university tertiary-care hospital, whose ED has a special circuit for the attention of chest pain patients, with similar features to the Barcelona chest pain unit. All Tenerife patients included in this study were ED patients with chest pain who were attended on this circuit.

Informed consent for urianalysis and participation in this study was obtained from all participants. Urine samples were stored at -80°C for subsequent analysis. Attending physicians had no information of drugs test results.

The following variables were studied: age, sex, outcome (death, hospital admission, discharge from ED), duration of ED stay for non-admissions, days of hospital stay and positive drugs test.

We measured in urine samples the levels of cocaine (benzoylecgonine and methylecgonine ether), cannabis (delta-9-tetrahidrocannabinol), amphetamine/metaamphetamine, opioids (morphine, and N-morphine). Drug detection was performed by semiquantative tests with fluorescence polarization immunoassay (FPIA)(AxSYM System, Abbott laboratories, Illinois, USA.). We considered the following values as positive: cocaine > 300 ng/ml, cannabis > 50 ng/ml, opioids > 300 ng/ml and amphetamine/metaamphetamine > 1000 ng/ml. Polyconsumption was defined as the presence of two or more drugs in the samples analyzed.

The project was approved by the local ethical research committee.

### Statistical analysis

Results for categorical variables are expressed as frequencies and percentages and 95% confidence intervals. Results for numerical and ordinal variables are expressed as means and standard deviations. Proportions were compared with Chi-square test or Fisher's exact test whenever required. Ranks between groups were compared with Mann-Whitney U test or Wilcoxon-Mann-Whitney test whenever required. A *P *value of less than 0.05 was considered to indicate statistical significance.

Statistical analysis was carried out with SPSS v. 14.0.1 (Chicago, ILL) and StatXact 5.0 (Cytel Co., Cambridge, MA).

## Results

Of 190 recorded patients, 140 agreed to participate in the study and complete information was obtained. There was some drug consumption in 15.7% (95% confidence interval: 9.6%–21.7%) and 6.4% (95% confidence interval: 2.0%–10.4%) showed cocaine-positive test. Polyconsumption was present in 4.3% of patients. Demographic features, ED management, and drug test results are shown in Table 1. There were differences between the two Hospitals in age, sex, hospital stay, cannabis consumption and polyconsumption.

**Table 1:** Demographic, ED Management and drug results by Hospital.

	**Total** n = 140	**Tenerife Hospital** N = 40	**Barcelona Hospital** n = 100	**P value**
**Age **(years)	58.76 ± 19.3	49 ± 15.6	63 ± 19.3	< 0.001
**Male sex **– no (%)	90 (65) [57–73]	32 (80) [67.6–92.4]	58 (59) [49–68]	0.019
**Emergency Dept. stay **(hours)	4.43 ± 6.1	4.56 ± 7.67	4.3 ± 4.93	0.99
**Hospital admission **– no (%)	55 (40) [32–49]	21 (53) [37–68]	34 (35) [26–45]	0.08
**Hospital stay **(days)	7.1 ± 6.5	5.9 ± 7.2	8.5 ± 5.3	0.01
**Cocaine** (positive test) – no (%)	9 (6) [2–10]	5 (12.5) [2.3–22.7]	4 (4) [1–8]	0.12
**Cannabinoids** (positive test) – no (%)	9 (6) [2–10]	6 (15) [3.9–26.1]	3 (3) [0–6]	0.016
**Opioids** (positive test) – no (%)	9 (6) [2–10]	2 (5) [0–11.7]	7 (7) [2–12]	0.5
**Amphetamines** (positive test) – no (%)	1 (1) [0–2]	0 (0) [0-0]	1 (0) [0-0]	0.99
**Any drug consumption **– no (%)	22 (15.7) [1–22]	9 (22.5) [9.6–35.4]	13 (13) [6–20]	0.099
**Polyconsumption **– no (%)	6 (4.2) [0.1–8]	6 (15) [3.9–26.1]	0 (0) [0-0]	< 0.001

We found an inverse relation between cocaine consumption and Hospital admission. One in two cocaine users also used cannabis. No differences were observed between cocaine users and non users regarding the concomitant use of opiods and amphetamines (Table 2). We found an association between cocaine and polyconsumption (p < 0.001).

**Table 2:** Demographics, ED management and other drug consumption in cocaine-positive/negative patients.

	**Total** n = 140	**Cocaine-positive Patients** n = 9	**Cocaine-negative Patients** n = 131	**P value**
**Age **(years)	58.76 ± 19.3	27.89 ± 5.77	60.89 ± 18.04	0.046
**Male sex **– no (%)	90 (65) [55–74]	9 (100) [100-100]	81 (62) [54–70]	0.017
**ED stay **(hrs)	4.43 ± 6.1	7.2 ± 2.3	4.13 ± 8.16	0.046
**Hospitalization **– no (%)	55 (40) [31–50]	0 (0) [0-0]	55 (42) [34–50]	0.008
**Hospital stay **(days)	7.1 ± 6.5	0 ± 0	7.6 ± 6.4	0.046
**Cannabinoids** (positive test) – no (%)	9 (6) [2–11]	5 (55.6) [23.1–88.1]	4 (3) [0–6]	< 0.001
**Opioids** (positive test) – no (%)	9 (6) [2–11]	0 (0) [0-0]	9 (7) [3–11]	0.54
**Amphetamines** (positive test) – no (%)	1 (0.7) [0–2]	1 (11.1) [0–31.6]	0 (0) [0-0]	0.99
**Polyconsumption **– no (%)	6 (4) [0–8]	6 (66.7) [35.1–96.9]	0 (0) [0-0]	< 0.001

Not unexpectedly, all cocaine-positive patients were young men, ranging in age from 22 to 34 years. With respect to follow-up data, all cocaine-positive patients were discharged home from ED after attention

## Discussion

In USA, with similar cocaine consumption rates to Spain, the Drug Abuse Warning Network DAWN estimates that cocaine was involved in 10% of drug misuse/abuse ED visits [8,9]. In the study of Hollander et al prevalence of cocaine use in chest pain of possible ischemic origin was 17%, ranged from 20% in Urban Hospitals ED to 7.45% in Suburban Hospital EDs [10].

With respect to other Spanish studies, our finding of 6.4% cocaine-positive chest pain patients was lower than the 25% reported by Sanjurjo et al [6,7]. This could be due to features of our study population who were patients with undisclosed cocaine-related chest pain, when in other series the patient visit was related with declared consumption or clinical toxic cocaine-related signs suggestive of consumption. As Perrone et al propose, drug screening for substance abuse in addition to clinical history is necessary for optimal identification of drug use in ED patients [11]. Our findings of low incidence of occult users added to those of other series in declared or suspected cases may provide a more realistic picture of cocaine consumption in these ED patients in Spain. According to the literature, it seems probable that the real incidence of cocaine use in non-traumatic chest pain patients is around 30% [6,7,10].

As in other studies, the great majority of our cocaine-consuming patients with chest pain were young people, in their third decade of life, and in general presumably at low risk of adverse cardiovascular events [10,12,13]. Thus none of them required admission to hospital. However, caution must be exercised when evaluating these patients with chest pain since there are no reliable tests to predict adverse cardiovascular outcomes in cocaine-associated chest pain [14].

The presence of cocaine in urine does not necessarily imply that this substance was the cause of the chest pain leading to their ED visit. Although urianalysis is usually positive in the first 48–72 hours alter consumption, chronic users can have positive urines for up to 2 weeks [15]. Our data on the prevalence of cocaine use in young people suggest that ED staff should be alert to possible consumption that is not disclosed by the patient. Junior doctors are less likely to routinely ask about cocaine use compared to other classical risk factors [5,16].

Despite the fact that we studied two demographically disparate groups of patients, we found no significant differences in clinical characteristics such as ED stay, need for hospitalization or length of hospital stay. Nor did we find differences in cocaine consumption between the two groups, but this could very well be explained by the small sample size. The high mean age of the Barcelona Hospital group probably accounts for the low number of cocaine-positive patients. In addition, this group was attended at the Chest Pain Unit, without any fast-track circuit patients (mostly young), as in previous studies by this group [6,7,17].

Further research with longer study periods and greater number of patients are required to confirm these findings.

## Conclusion

This study found undisclosed cocaine consumption in 6.4% (95% confidence interval: 2.0%–10.4%) of adult patients presenting at Emergency Department for chest pain. This finding in chest pain patients who consented to urine analysis suggests that the true incidence of cocaine use leading to such ED visits may be higher.

## Competing interests

The authors declare that they have no competing interests.

## Authors' contributions

GB, MS and OM were responsible for study design, analyzing and interpretation data. BL, MG, EV and AD participated in collecting data. JB carried out the immunoassays. AJ performed the statistical analysis and interpretation data. Al the authors read and approved the final manuscript.
